# ISSUES IN HOME CARE AND CAREGIVING ARE MEDICAL FOSTER HOMES SERVING VETERANS WHO HAVE SIMILAR FUNCTIONAL IMPAIRMENT AS TYPICAL NURSING HOME RESIDENTS?

**DOI:** 10.1093/geroni/igz038.3032

**Published:** 2019-11-08

**Authors:** Cari Levy, Kate Magid, Chelsea Manheim, Kali S Thomas, Leah M Haverhals, Debra Saliba

**Affiliations:** 1 VA Rocky Mountain Regional VA Medical Center, Aurora, Colorado, United States; 2 Seattle/Denver Center of Innovation, VA Eastern Colorado Health Care System, Aurora, Colorado, United States; 3 Rocky Mountain Regional Medical Center, Aurora, Colorado, United States; 4 Providence VA Medical Center, Providence, Rhode Island, United States; 5 Seattle-Denver Center of Innovation, Denver, Colorado, United States; 6 RAND Corporation, Santa Monica, California, United States

## Abstract

The Veterans Health Administration’s (VHA’s) Medical Foster Home (MFH) program was developed as a community-based alternative to institutional care. This study compares the clinical and functional characteristics of Veterans in the VHA MFH program to residents in nursing homes to understand whether MFHs substitute for nursing home care or serve a population with different care needs. All data were derived from Minimum Data Set (MDS) 3.0 assessments. Nurses collected MDS assessments from Veterans (n=92) in 4 MFHs between April 2014-December 2015. Data for nursing home residents were from a national nursing home dataset of residents with an annual MDS assessment in 2014 (n=818,287). We found that MFH Veterans were more likely to be male, have higher functional status, and perform more activities of daily living (ADLs) independently relative to nursing home residents (p<0.01 for all comparisons). Yet, a similar proportion of MFH Veterans and nursing home residents required total assistance in 9 of the 11 measured ADLs. Cognitive impairment, neurological comorbidity, and psychiatric comorbidity were similar in both cohorts; however, MFH Veterans were more likely to have traumatic brain injury (p<0.01), higher Patient Health Questionnaire (PHQ)-9 depression scores (p=0.04) and less likely to have anxiety (p=0.05). Our results suggest there are two distinct MFH populations, one with lower-care needs and another with Veterans completely dependent in performing ADLs. Given these findings, MFHs may be an ideal setting for both low-care nursing home residents with less functional impairment as well as residents with higher care needs who desire community-based long-term care.

